# Platelet-Derived Microvesicles in Cardiovascular Diseases

**DOI:** 10.3389/fcvm.2017.00074

**Published:** 2017-11-21

**Authors:** Maria T. K. Zaldivia, James D. McFadyen, Bock Lim, Xiaowei Wang, Karlheinz Peter

**Affiliations:** ^1^Atherothrombosis and Vascular Biology, Baker Heart and Diabetes Institute, Melbourne, VIC, Australia; ^2^Department of Medicine, Monash University, Melbourne, VIC, Australia; ^3^Department of Haematology, The Alfred Hospital, Melbourne, VIC, Australia; ^4^Heart Centre, The Alfred Hospital, Melbourne, VIC, Australia

**Keywords:** microvesicles, platelet-derived microvesicles, cardiovascular disease, therapeutic potential, hemostasis, inflammation, angiogenesis

## Abstract

Microvesicles (MVs) circulating in the blood are small vesicles (100–1,000 nm in diameter) derived from membrane blebs of cells such as activated platelets, endothelial cells, and leukocytes. A growing body of evidence now supports the concept that platelet-derived microvesicles (PMVs), the most abundant MVs in the circulation, are important regulators of hemostasis, inflammation, and angiogenesis. Compared with healthy individuals, a large increase of circulating PMVs has been observed, particularly in patients with cardiovascular diseases. As observed in MVs from other parent cells, PMVs exert their biological effects in multiple ways, such as triggering various intercellular signaling cascades and by participating in transcellular communication by the transfer of their “cargo” of cytoplasmic components and surface receptors to other cell types. This review describes our current understanding of the potential role of PMVs in mediating hemostasis, inflammation, and angiogenesis and their consequences on the pathogenesis of cardiovascular diseases, such as atherosclerosis, myocardial infarction, and venous thrombosis. Furthermore, new developments of the therapeutic potential of PMVs for the treatment of cardiovascular diseases will be discussed.

## Introduction

Extracellular vesicles (EVs) encompass a broad range of vesicles released from cells (1). EVs can be classified into different subsets according to their size, cellular origin, content or the mechanism leading to their formation (Table 1). Microvesicles (MVs)—also referred to as microparticles—are vesicles typically around 100–1,000 nm in size. By contrast, smaller vesicles (30–100 nm) are referred to as exosomes, while larger vesicles containing nuclear materials are referred to as apoptotic bodies. Although there is a general consensus in most studies that apoptotic bodies are particles >1 μm (2, 3), there are several studies that describe apoptotic bodies to have a smaller size range of 0.5 µm (4, 5).

**Table 1:** Characteristics of extracellular vesicles.

	Exosome	Microvesicle	Apoptotic body
Size	≈20–100 nm	≈0.1–1 µm	>1 µm

Origin	Multivesicular bodies, internal compartments	Plasma membrane	Cellular fragments

Markers	– Tetraspanins (CD63, CD9, and CD81) – ALG-2-interacting protein X – Tumor susceptibility gene 101 protein – Heat shock 70-kDa proteins – Major histocompatibility complex class I and class II	– Phosphatidylserine (PS) – Integrins – Selectins – CD40 ligands – Other antigens of parental cell	– Histones – Fragmented DNA – PS

Reference	– (6–9)	– (9–14)	– (9, 15, 16)

In the context of platelet biology, the plasma membrane fragments shed from activated platelets initially observed to possess procoagulant function were described as “platelet dust” by Wolf (17). Subsequent studies employing electron microscopy demonstrated the budding of vesicles from the platelet plasma membrane (18) thus confirming the cellular origin of the fragments detected by Wolf (17). In fact, 60–90% of EVs have been shown to be derived from platelets as indicated by positive CD41 staining (19). Since then, the role of EVs in the field of cardiovascular research has garnered a huge amount of interest due to their putative role in various pathological conditions.

Elevated levels of platelet-derived microvesicles (PMVs) are observed in diabetes mellitus, sepsis, rheumatoid arthritis, vascular inflammation, and cardiovascular diseases (20–31). Indeed, the pathological events associated with these diseases activate platelets (32–35), which have been demonstrated to increase PMV release, while at the same time, a subpopulation of PMVs coming from agonist-activated platelets have been demonstrated to contribute to pathological events (36). Thus, PMVs may well be both, one of the causes and a consequence of the pathophysiology that drives various diseases.

This review will focus on our current understanding of PMVs in mediating hemostasis, inflammation, and angiogenesis, which are all factors contributing to the pathogenesis of cardiovascular diseases. Furthermore, the clinical relevance of PMVs will be discussed in the context of their therapeutic potential in the treatment of cardiovascular diseases.

## Formation and Clearance of PMVs

**Figure 1:**
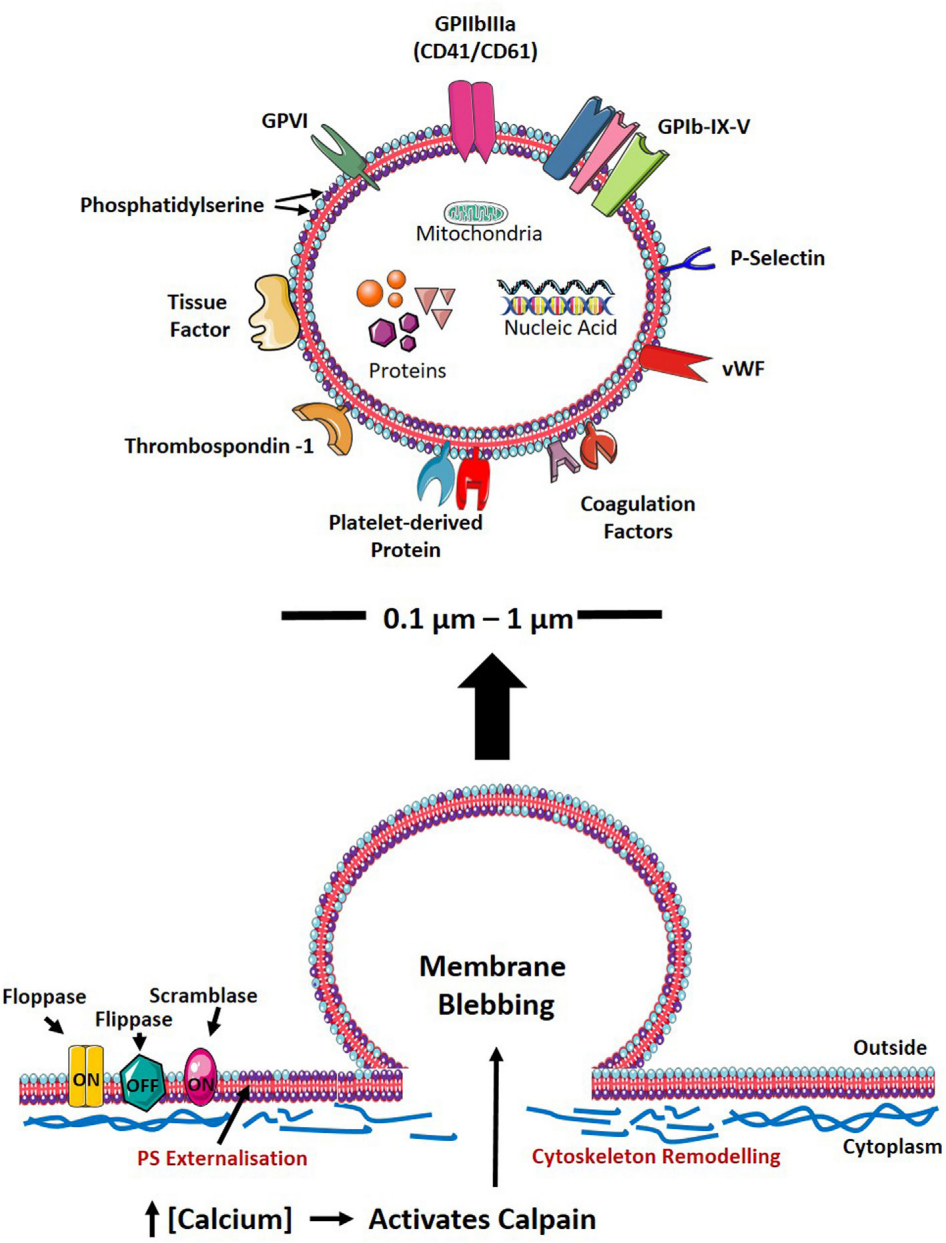
PMV formation and characteristics. Upon cellular activation, the elevation of intracellular calcium inhibits flippase, while activating floppase and scramblase (TMEM16F), mediating the externalization of negatively charged PS (indicated as purple phospholipid). Furthermore, increased intracellular calcium leads to reorganization of the cytoskeleton by activating calpain, thereby cleaving PMVs and releasing them into the circulation. The size, physical characteristics, and cargo of PMVs depend on the environment and agonist(s) causing PMV generation. PMVs share many surface proteins with platelets such as integrins, selectins, adhesion receptors, coagulation factors, and other platelet-derived proteins. PMVs are packed with proteins including growth factors, cytokines/chemokines, and apoptotic proteins. PMVs also carry nucleic acids (mRNA, miRNA, and RNA) and mitochondria. PS, phosphatidylserine; GP, glycoprotein; vWF, von Willebrand factor; RNA, ribonucleic acid; mRNA, messenger RNA; miRNA, microRNA; PMV, platelet-derived microvesicle.

Platelet-derived microvesicle formation is complex, and the exact mechanisms involved in the generation of PMVs to date remains to be clearly defined. However, it has been demonstrated that the generation of PMVs can be triggered by various mechanisms: (1) *via* platelet activation by soluble agonists or (2) shear stress or (3) glycoprotein (GP) IIb/IIIa outside-in signaling. In the case of platelet activation in response to soluble agonist stimulation or in response to high shear stress in the vasculature (37–39), sustained elevation of intracellular calcium has been observed, which initiates the loss of lipid asymmetry of the plasma membrane and cytoskeletal reorganization, ultimately leading to PMV generation (Figure 1) (40). The exposure of negatively charged phospholipids, such as phosphatidylserine (PS), on the outer leaflet of the plasma membrane is regulated by the calcium-dependent scramblase transmembrane protein (TMEM16F) (41). Likewise, calpain, a calcium-dependent protease, is central to regulating cytoskeletal reorganization thus facilitating PMV shedding (42). By contrast, unstimulated platelets have been demonstrated to generate PMVs *via* GPIIb/IIIa signaling, which destabilizes the actin cytoskeleton, resulting in shedding of PMVs in the absence of soluble agonist stimulation (43). Once generated, PMVs have generally been observed to have a relatively short lifespan with studies demonstrating PMVs to have half-lives of 30 min in mice (44), 10 min in rabbits (45), and approximately 5.5 h in apheresis-derived PMVs (46). Active endocytosis has been demonstrated to be involved in the clearance of MVs (47) including those of PMVs (48). Indeed, several studies demonstrated the involvement of PS-dependent phagocytic processes in the clearance of PMVs in the circulation (44, 49–52). Several opsonins such as complement component C3b, β2-glycoprotein-1, lactadherin, and developmental endothelial locus-1 facilitate this PS-dependent phagocytosis (44, 49–51, 53). Upon engulfment of EVs by phagocytic cells such as macrophages and dendritic cells, liver X receptor (LXR) and peroxisome proliferator-activated receptor pathways are activated, which both are known to be induced by lipid derivatives (54). Indeed, the activation of the cholesterol derivative-sensitive pathway LXR by PMVs has been previously demonstrated in plasmacytoid dendritic cells (pDCs) (52), thereby highlighting the importance of lipid composition of EVs on the target cell responses after engulfment. The internalization of PMVs is not only essential for the clearance of PMVs but also ensures the delivery of the MV content into the target cell and thereby exerting their effector functions. Although studies have shown PS-dependent phagocytosis to be involved in the clearance of PMVs, other mechanisms are also involved in this process, which have previously been reviewed in detail by Mulcahy et al. (55).

## Composition of PMVs

From the humble origin of being just “platelet dust,” it is now apparent that PMVs can mediate a diverse range of physiological responses. Their capacity to exert their biological role is attributed to their cell membrane composition and molecular cargo. The phospholipid composition of PMVs is a composite of the platelet plasma and granule membranes with high cholesterol content, also indicating an enrichment of lipid rafts (35). PMVs share many of the antigens as their parental cells that regulate cell adhesion, activation, and coagulation reactions (Figure 1) (10–13). These include various GPs, tissue factors (TF), selectins, and coagulation factors V and VIII (10–13). PMVs are packed with numerous biological molecules, which facilitate the transportation and delivery of bioactive mediators that can modulate the function of target cells. PMVs carry cytokines and chemokines such as interleukin (IL)-1β, CXCL4, CXCL7, and CCL5 (11, 56). In addition, a vast amount of proteins, growth factors, and genetic material such as ribonucleic acid (RNA), messenger RNA, and microRNA can be packaged into PMVs (12, 57–60). Moreover, PMVs have recently been described to carry mitochondria, which can influence inflammatory responses (61).

The mechanisms of selective packaging have been demonstrated widely in EVs (62–65). Albeit not fully characterized in PMVs, the difference in lipid composition between PMVs and their parental cell, platelets (35), provide evidence for selective membrane assembly. Interestingly, it has been observed that the physical and biological components of PMVs are influenced by the stimulus used to generate PMVs (66–69). The heterogeneity observed in PMVs may explain why they have a diverse range of biological roles. For instance, larger PMVs may be enriched with more receptors and contents within, and thus can exert more effects, while smaller PMVs can deliver their biological cargo to areas that are otherwise difficult to enter, such as tumors, or to cross, such as the blood–brain barrier. Overall, it is important to understand that various pathological conditions will produce different types of PMVs carrying unique biological cargos that exert specific effects on targets cells. However, the precise mechanism by which PMVs selectively package and release their biological cargo to influence cellular function needs to be carefully determined in the future.

## Biological Function of PMVs

### Hemostasis

It is perhaps not surprising that PMVs are most widely recognized for their role in mediating hemostasis given the resemblance in lipid composition and biological cargo they share with their parent cell—the platelet. Diseases that affect PMV shedding in the circulation have provided insight into their ability to regulate hemostasis. For example, patients with Castaman’s defect, where platelets have an isolated inability to generate MVs display a bleeding phenotype (70). Similarly, Scott syndrome, where platelets cannot externalize PS and generate PMVs, is associated with a marked bleeding diathesis (41, 70–72). These disorders highlight the importance of PMVs in mediating hemostasis.

In accordance with their ability to regulate hemostasis, PMVs display both pro- and anticoagulant properties. The assembly of the tenase and prothrombinase complexes, and thus thrombin generation, is increased in the presence of PMVs due to the PS in the outer leaflet of the cell membrane (73). PMVs also express TF, which can initiate the extrinsic coagulation pathway by activating Factor VII (73, 74). On the contrary, PMVs have anticoagulant activity, which is associated with the binding of the natural anticoagulant, protein S and activation of protein C (75–79). Given the potential pro- and anticoagulant effects of PMVs, the tight regulation of PMV levels is likely an important factor regulating the hemostatic response.

### Inflammation

Akin to the wealth of literature demonstrating an important pro-inflammatory role for platelets, there is now a growing body of evidence demonstrating that PMVs can regulate inflammatory responses. The immunoglobulins, antigens, cytokines, and chemokines that PMVs carry can directly regulate immune responses (11, 56, 80). The pro-inflammatory effects of PMVs can be demonstrated through their interactions with monocytes and neutrophils. Mechanistically, PMVs bind to leukocytes and form aggregates and can induce monocytes to release inflammatory mediators including IL-1β, tumor necrosis factor (TNF)-α, monocyte chemoattractant protein-1, and matrix metalloproteinase (MMP)-9, which enhance monocyte migration (81, 82). Lipopolysaccharide-induced PMVs carry IL-1β in its mature form, which can activate endothelial cells and induce leukocyte adhesion thus promoting endothelial inflammation (56). Furthermore, mitochondria that are released *in vivo* in sterile inflammatory diseases, such as rheumatoid arthritis, have been observed to be packaged within PMVs, which can be hydrolyzed by phospholipase A2 IIA to generate bioactive mediators which promote neutrophil pro-inflammatory responses (61).

Intriguingly, there are reports that PMVs can also act as anti-inflammatory mediators. Recently, Dinkla and colleagues (83) have shown that PMVs prevent the differentiation of regulatory T cells into a pro-inflammatory phenotype. PMVs can bind to CCR6-HLA-DR^+^ regulatory T cell subsets *via* P-selectin and inhibit the production of IL-17 and interferon- γ (83). In accordance, PMVs from platelet concentrates can modify innate immune cells such as macrophages and dendritic cells. Macrophage activation is attenuated in the presence of PMVs as indicated by reduced production of TNF-α and IL-10 (84). PMVs also alter the function of monocyte-derived dendritic cells as demonstrated by their reduced capacity to present antigens, diminished production of pro-inflammatory cytokines and decreased phagocytic activity (84). PMVs can also modify inflammatory effects of the target cell. For instance, PMVs have been demonstrated to regulate the inflammatory responses of mast cells by the transfer of Lipoxygenase 12 (85). This leads to the production of Lipoxin A4, which induces anti-inflammatory and anti-angiogenic responses on endothelial cells by suppressing the generation of pro-inflammatory cytokines (85, 86). Furthermore, pDCs, a subset of dendritic cells that augment inflammatory processes by producing a large amount of pro-atherogenic type 1 interferons were observed to engulf PMVs in a PS-dependent manner (52). PMVs were observed to inhibit pDCs pro-inflammatory response by reducing the secretion of TNF-α and IL-8, signifying an anti-inflammatory mechanism of PMVs (52).

In addition, our group has demonstrated that pentameric protein C-reactive protein (pCRP) binds to different MVs including PMVs (87). These pCRP-MVs, albeit not pro-inflammatory in healthy individuals, can aggravate existing tissue injury by activating the classical complement pathway and enhancing leukocyte recruitment to inflamed tissues (87). MVs not only bind pCRP but also convert pCRP to a highly pro-inflammatory monomer of C-reactive protein (mCRP), which can bind to endothelial cells and generate pro-inflammatory signals (88–91). In addition, autoantigen proteinase 3, an elastin degrading protease, binds to PS expressing MVs, thereby promoting inflammation *via* the generation of reactive oxygen species in neutrophils (92). Thus, these studies further highlight the ability of PMVs to partner with proteins to induce a pro-inflammatory phenotype. Therefore, PMVs may play a dual role in inflammation as they may instigate either pro- or anti-inflammatory responses depending on the cell membrane composition and biological cargo transported by the PMV.

### Angiogenesis

In addition to harboring a number of pro-inflammatory cytokines, PMVs may carry growth factors such as vascular endothelial growth factor (VEGF), fibroblast growth factor 2, and lipid growth factors suggesting PMVs may play an important role in regulating angiogenesis (93). In accordance, Kim and colleagues have demonstrated that PMVs can inhibit apoptosis while enhancing cell migration, proliferation, survival, and tube formation in human umbilical vein endothelial cells (93). PMVs may also enhance pro-angiogenic MMP-2 and MMP-9 expression in endothelial cells *in vitro* and *in vivo* (94). Furthermore, PMVs stimulate the growth of endothelial progenitor cells, thus contributing to the formation of new blood vessels (93).

The role of PMVs in regulating angiogenesis in the context of cardiovascular diseases has been highlighted by Brill and colleagues (95). The authors established that PMVs can induce angiogenesis *in vitro* caused by cytokines, VEGF, basic fibroblast growth factor, and platelet-derived growth factor packaged within PMVs in a process linked to Src, PI-3K, and ERK signaling (95). Moreover, the injection of PMVs in ischemic heart muscle induces the formation of blood vessels in a murine model of myocardial infarction (MI), signifying PMVs capability to induce myocardial angiogenesis in the setting of ischemia (95). PMVs have also been shown to facilitate endothelial repair after arterial injury by enhancing the vasoregenerative capacity of early outgrowth cells (EOCs) (96). PMVs enhance the recruitment, migration, and differentiation of EOCs at the site of injury by enhancing angiogenic growth factors that stimulate resident mature endothelial cells (96). The ability of PMVs to induce angiogenesis has also been demonstrated in the context of neurogenesis following brain injury and tumor progression (97, 98) thus, highlighting the potentially broad role of PMVs in endothelial repair and angiogenesis.

## PMVs in Cardiovascular Diseases

### Atherosclerosis

The rupturing of an atherosclerotic plaque can lead to MI and stroke, which are leading causes of death and disability globally. Indeed, excessive amounts of PMVs have been observed in patients with atherosclerosis (23–28). The increase in PMV numbers was found to correlate with multiple parameters including carotid artery intima media thickness, lipid-rich atherosclerotic plaques, and plaque burden (23–27). In the setting of atherosclerosis, increased hemodynamic shear stress due to plaque-associated luminal stenosis as well as the accumulation of oxidized low-density lipoprotein can activate platelets and stimulate generation of pro-inflammatory PMVs (33, 99). In atherosclerosis, monocytes adhere to activated endothelial cells, infiltrate the intima, and differentiate to tissue macrophages, which then engulf lipids and form foam cells (100). Smooth muscle cells migrate from the media to the intima and produce extracellular matrix, forming the fibrous cap (100). Macrophages and smooth muscle cells can undergo apoptosis leading to accumulation of extracellular lipid which forms the necrotic core (100). Indeed, PMVs have been implicated with these different stages of atherogenesis. For instance, PMVs encapsulate and transport miR-223 to endothelial cells, which can trigger endothelial apoptosis *via* the insulin-like growth factor-1 receptor (60). PMVs, together with endothelial MVs, increase endothelial permeability thereby influencing vascular endothelial dysfunction, an early step in the development of atherosclerosis (101). The presence of P-selectin expressed by PMVs allows them to interact with leukocytes *via* P-selectin GP ligand-1 thereby facilitating leukocyte accumulation at the site of endothelial injury and enhancing leukocyte infiltration from the blood vessel to the intima (82). Further to this, PMVs can transfer the pro-atherogenic cytokine RANTES to endothelial cells and induce monocytes and endothelial cells to release pro-inflammatory cytokines such as IL-8, IL-1β, TNF-α, and IL-6, further enhancing leukocyte adhesion and infiltration (102–104). PMVs encapsulate active caspase-3 that can induce macrophage apoptosis (105). As a consequence in atherosclerosis, lipids derived from dead macrophages can accumulate and can contribute to the formation of the necrotic core. Furthermore, PMVs have been demonstrated to stimulate smooth muscle cells, leading to the migration of smooth muscle from the media to the intima thereby enhancing lesion progression (106, 107).

However, the main caveat of these studies is the fact that PMVs are only detected in the circulating blood of patients with atherosclerosis but are not found in the atherosclerotic plaque itself (108). This raises the question as to whether PMVs are merely associated with atherogenesis or play an active role in disease pathogenesis. The absence of PMVs in the plaque, while intriguing because of the evidence of PMVs’ infiltration in other inflamed tissues, such as arthritic joints (36), could suggest selective removal of PMVs as they engage with other cells and exert their effects at the intraluminal area of the vessel in the setting of atherosclerosis. PMVs are highly subjected to endocytosis/phagocytosis, due to the high expression of adhesion molecules and PS (109). This potentially enhances the clearance of PMVs in comparison with other blood stream-derived MVs, which may possibly be one of the mechanisms contributing to the absence of PMVs in the atherosclerotic plaque. These are important questions for further studies.

### Acute Coronary Syndromes (ACS)

High levels of circulatory PMVs have been observed in patients with ACS and are also associated with the degree of elevation of cardiac enzymes, IL-6, and CRP levels (29–31). The elevated levels of PMVs in the plasma of patients with ACS persisted for up to 4 years after MI and is linked with markers of coagulation activation and soluble CD40L (110). In accordance with these findings, PMVs have been demonstrated to correlate with the size of myocardium at risk and microvascular dysfunction after ST elevation MI (STEMI) (111, 112). Also, it appears likely that MVs play an active role in promoting vascular inflammation and cardiac damage in patients after an ACS since MVs, including PMVs containing pro-inflammatory isoforms of CRP, have been demonstrated to be elevated in these patients (87, 88). Furthermore, PMVs independently predict future admission for major bleeding in non-STEMI patients (113). Taken together, these data suggest that PMVs detected in patients with ACS may also act as reporters of vascular inflammation, microvascular obstruction and myocardial damage in cardiovascular diseases (29, 30, 111, 112). While elevated levels of PMVs are often observed in patients with ACS, there are few studies that have reported variances in the levels of PMVs and the lack of association of PMVs with the severity of coronary artery disease (114, 115). The discordance in the levels of PMVs in ACS may be due to the variability of inclusion criteria and medication of the patients enrolled in each study. Also, the lack of consensus in the characteristic and definition of MVs may account for the variations observed in the literature. Indeed, further studies are required, with establish common protocols and clear MVs nomenclature, to fully elucidate the role of PMVs in ACS.

### Thrombotic Disorders

Thrombotic complications are often observed in patients with cardiovascular diseases. Augmented shedding of PMVs is deleterious and may contribute to thrombosis. For instance, in patients with immune thrombocytopenia, there are high levels of PMVs despite low platelet counts, which have been linked to a paradoxical increased risk of thrombotic events (116, 117). Increased PMVs are also detected in other thrombotic diseases such as acute pulmonary embolism and deep vein thrombosis (DVT) (118–121). Likewise, PMVs have been detected to be elevated in the context of thrombophilic states such antithrombin deficiency, protein C deficiency, and the Factor V Leiden mutation—all predispositions to venous thrombosis (122, 123). Increased levels of PMVs are observed to be associated with a heightened risk of venous thromboembolism, and PMVs have been proposed as a biomarker to help diagnose patients with DVT (119, 121). Similar to ACS, variation in plasma levels of PMVs are observed in thrombotic disorders (122, 124–126). Inconsistencies in processing blood samples such as handling, storage and methodology used in isolating PMVs may cause artifactual generation of PMVs and represent one of the underlying reasons for the discrepancies observed in the level of PMVs. Therefore, further studies are needed to fully unravel the role of PMVs in thrombotic diseases.

## Current Challenges in EV Research

Despite the remarkable progress in the field of EVs, clinical translation of EVs as a diagnostic or prognostic marker of pathological states remains a challenge (127). To fully unravel the potential of EVs in the diagnosis and therapy of cardiovascular diseases, it is imperative to understand the biological roles of PMVs *in vivo*. One of the drawbacks in the field of EVs is that most of the experiments demonstrating the physiological effects of EVs on target cells, such as endothelial cells, have been done *in vitro* in culture. The key weakness of this approach is that endothelial cells under *in vivo* conditions are under constant steady-state exposure to EVs. It is not clear how the response of cells in the *in vitro*, which is typically EV-free, to the sudden exposure of EVs is related to the *in vivo* setting. Furthermore, due to the lack of common practice in sample preparation and analysis, EV counts and phenotypes may vary dramatically between laboratories, making data analyses and clinical translation difficult. Indeed, several studies have highlighted the effects of pre-analytical variables on EV measurements. These include the type of anticoagulant used in collecting blood samples, centrifugation protocol, the storage of samples and staining protocols used for surface membrane antigens for determining the cellular origin of EVs (128–131). In regard to PMVs, preventing platelet activation and ensuring complete removal of platelets during processing of samples are crucial as this may result in inaccurate findings. It is also essential to be mindful of the storage and thawing conditions of samples as this leads to changes in the number of PMVs and Annexin V binding (128–130). In addition to this variability, sample handling, isolation protocol, different antigens used to determine cellular origin, inclusion criteria for patients, and their clinical characteristics may account for the qualitative and quantitative variations observed in the literature regarding the characterization of EVs.

The technological advancements have facilitated new methods to improve the purification and detection of EVs from biological fluids (1, 127, 132–135). These include the following: sensitive single particle detection devices (tunable resistive pulse sensing; nanoparticle tracking analysis; and dynamic light scattering), flow cytometry (conventional; imaging; and impedance based), proteomics, and atomic force microscopy (1, 127, 130, 134, 136–138). However, a combination of multiple methods is still necessary to assess both physical and biological properties of EVs (1, 135). In light of the current limitation of EV studies, the International Society of Extracellular Vesicles endeavors to provide guidelines, harmonizing nomenclatures and practices in an effort to improve reproducibility of EV experiments and to eliminate ambiguity in the field of EVs (139–141). Furthermore, this society has already published several position papers in the Journal of Extracellular Vesicles (139–142) and provides public online databases that catalog EV-associated components, thereby assisting researchers to optimize their practices (EV-TRACK, ExoCarta, Vesiclepedia, and EVpedia).

## Therapeutic Potential of PMVs

Given the role of PMVs in cardiovascular diseases, this raises the question as to whether MVs can be exploited for therapeutic benefit. Indeed, pharmacological studies that alter the levels of PMVs in the circulation have shown encouraging results (143–146). Also, the physical and biochemical properties of PMVs are advantages that can be utilized for the purpose of developing a therapy for cardiovascular diseases (Table 2). For instance, PMV-inspired nanovesicles have been engineered to deliver thrombolysis specifically to sites of clot formation (147). The design of the therapeutic nano vesicle was based on the biological aspects of PMVs that are relevant to thrombus formation. Surface receptors GPIIb/IIIa and P-selectin were used to target the site of clot formation, and the enzyme phospholipase A2 was employed to rupture the vesicle and release the lytic drug, thus allowing the targeting of thrombolysis only at sites of clot formation (147). Thus, this study highlights the feasibility of altering the cargo of EVs and surface receptors for site-specific delivery (Table 2). Also, natural PMVs have showed therapeutic potential to treat cardiovascular diseases. Pretreating circulating-angiogenic cells (CACs) with PMVs derived from atherosclerotic patients (PMV-CACs) enhances the re-endothelization capacity of these cells (148). PMV-CACs enhance blood flow and increase capillary density in rats suffering from hind limb ischemia *via* PMV release of RANTES (148). Furthermore, a cardioprotective role of MVs, specifically PMVs, has been demonstrated in a study by Ma and colleagues (149). In this study, the transfusion of PMVs from rats that underwent hind limb ischemia–reperfusion preconditioning resulted in increased levels of PMVs and a reduction of infarct size, indicative of a protective role of PMVs in the context of cardiac ischemia–reperfusion injury. This was further supported by data showing that PMVs transfused into rats with middle cerebral artery occlusion reduced infarct area (150). Similarly, other types of EVs have shown promising therapeutic potential. EVs, mainly MVs and exosomes, displayed a cardioprotective role by enhancing the recovery of cardiac function in a postinfarct heart failure animal model (151). Although the precise mechanisms involved in the protective role of EVs in cardiovascular diseases is still incompletely understood, the capacity of EVs to carry vast biological cargos and their ability to transfer a wide array of bioactive molecules to target cells may explain the beneficial effect of EVs as a therapy for cardiovascular diseases. Furthermore, EVs can be used as a drug delivery system to increase solubility, stability, and bioavailability of hydrophobic drug in the blood circulation (Table 2). Sun et al. encapsulated the drug curcumin in exosomes and successfully delivered the drug to activated monocytes, thus inducing an anti-inflammatory response in a preclinical model of septic shock (152). Despite the early promise from these studies involving PMVs, further studies are needed to delineate the potential utility of PMVs in cardiovascular diseases. Further to this, a number of challenges remain in translating therapeutic EVs to the clinic (Table 2).

**Table 2:** Advantages and disadvantages of extracellular vesicles (EVs) as emerging therapy for cardiovascular diseases.

Advantages	Disadvantages
Natural homing ability and specific transfer of bioactive molecules (152, 153)	Lack of standardization of pre-analytical variables (127, 140)
Highly hydrophobic drugs and hydrophilic drugs can be packaged (152, 154)	No clear nomenclature leading to variable qualitative and quantitative analysis (1, 155)
Good delivery vehicle for drugs based on the ability to cross blood–brain barrier (153, 156)	No recommended isolation protocol for clinical grade production and quality control of EV-based therapeutics (68, 142)
Easy to adapt/optimize content and surface receptors for site-specific delivery (147, 154, 156)	Comprised of heterogeneous components depending on the isolation, handling, and agonist(s) used (67, 69, 128–130)
More stable upon freezing and thawing compared with cells, biocompatible, and non-cytotoxic (142, 156)	The need to establish techniques and methodologies to rigorously quantify and characterized the molecular and physical aspects of EVs (142)

## Conclusion

**Figure 2:**
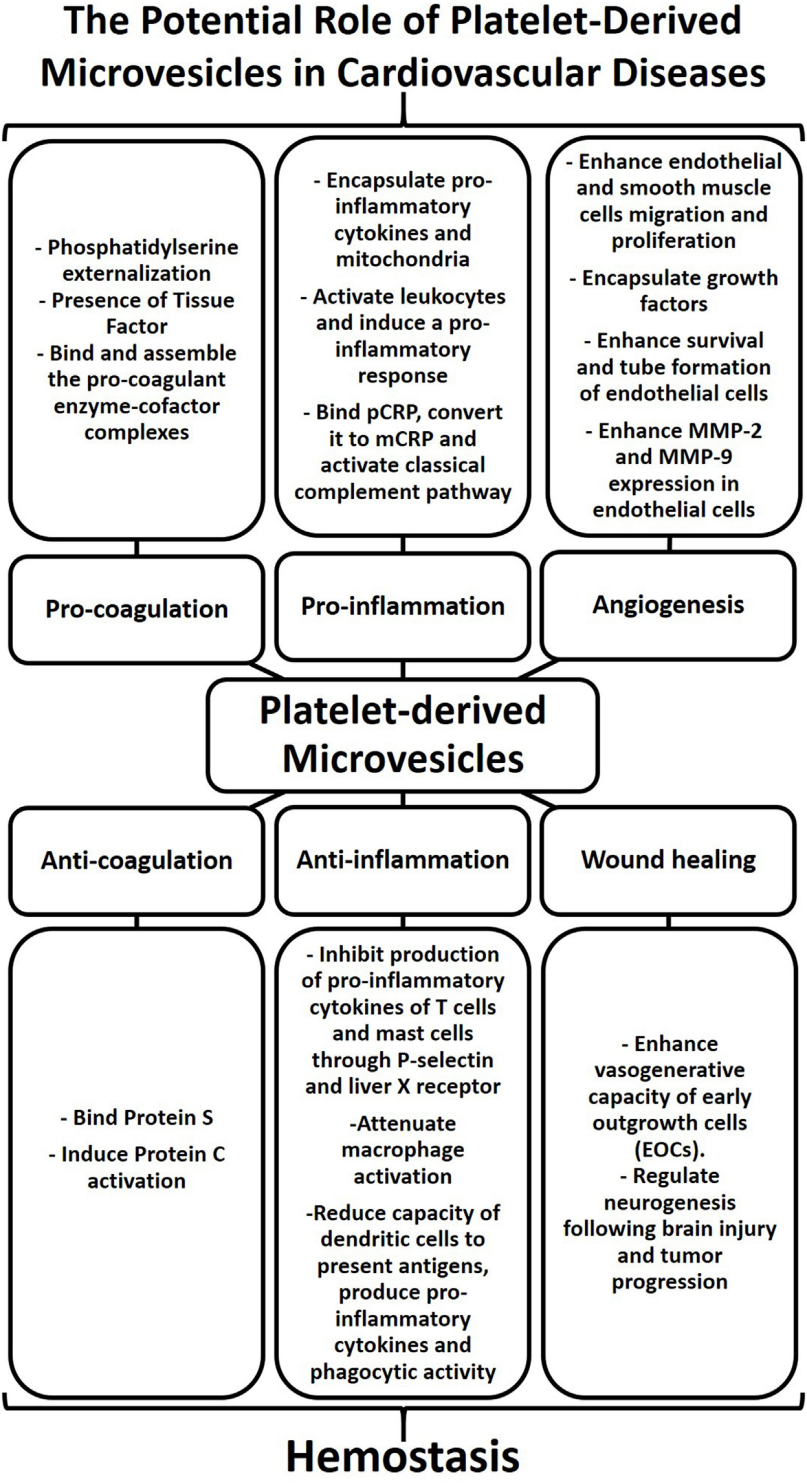
Platelet-derived microvesicles (PMVs) as regulators of hemostasis and contributors to cardiovascular diseases. The physical characteristics of the cell membrane and biological cargo define the biological role of PMVs. PMVs bind natural anticoagulants thereby preventing coagulation. PMVs can also inhibit cytokine production leading to a reduction of vascular inflammation. In addition, PMVs can enhance the vasogenerative capacity of cells, thus highlighting their role in wound healing. While PMVs play a major role in regulating hemostasis, excessive numbers of PMVs can also contribute to cardiovascular diseases. The presence of phosphatidylserine and tissue factor in PMVs can induce procoagulant enzyme–cofactor complexes that favor thrombosis. PMVs can also induce cytokine production, bind protein C-reactive protein (pCRP), and convert it to monomer of C-reactive protein (mCRP), thereby promoting inflammatory responses. The activation of smooth muscle cells, endothelial cells, and leukocytes by PMVs as well as growth factors encapsulated within PMVs can stimulate angiogenesis. Therefore, PMVs may stimulate or dampen coagulation, inflammation, and angiogenesis and may thereby contribute to cardiovascular diseases.

Platelet-derived microvesicles have indeed come a long way from their initial descriptions as “platelet dust” to now being considered as major mediators of intercellular communication. Their role in hemostasis, inflammation, angiogenesis, and wound healing may be beneficial or deleterious and can contribute to the pathogenesis of cardiovascular diseases (Figure 2). PMVs represent a heterogeneous population of EVs derived from platelets. This heterogenicity is mainly due to the variability of stimuli capable of inducing platelet activation and PMV release. Indeed, the mode of platelet activation seems to define the size, content, and amount of PMVs, which together dictate their fate (cell targeting or not) and their effects and potential involvement in diseases. However, despite the undoubted progress in our understanding of the biological roles PMVs, there remains a pressing need to establish common protocols, analysis, and nomenclature in the field of EV research. Advancing our knowledge of the biological function of EVs holds promise to influence the treatment of cardiovascular diseases. Most interestingly, EV-based therapy represents an emerging and novel biological therapeutic approach for cardiovascular diseases. Therefore, harnessing our knowledge of EV biology may indeed unlock the full potential of EVs for the diagnosis and therapy of cardiovascular diseases and beyond.

## Author Contributions

All authors listed have made a substantial, direct, and intellectual contribution to the work and approved it for publication.

## Conflict of Interest Statement

The authors declare that the research was conducted in the absence of any commercial or financial relationships that could be construed as a potential conflict of interest.
